# National, Regional, State, and Selected Local Area Vaccination Coverage Among Adolescents Aged 13–17 Years — United States, 2017

**DOI:** 10.15585/mmwr.mm6733a1

**Published:** 2018-08-24

**Authors:** Tanja Y. Walker, Laurie D. Elam-Evans, David Yankey, Lauri E. Markowitz, Charnetta L. Williams, Sarah A. Mbaeyi, Benjamin Fredua, Shannon Stokley

**Affiliations:** ^1^Immunization Services Division, National Center for Immunization and Respiratory Diseases, CDC; ^2^Division of Viral Diseases, National Center for Immunization and Respiratory Diseases, CDC; ^3^Division of Bacterial Diseases, National Center for Immunization and Respiratory Diseases, CDC; ^4^Leidos Health, Inc., Reston, Virginia.

## Abstract

The Advisory Committee on Immunization Practices (ACIP) recommends routine vaccination of persons aged 11–12 years with human papillomavirus (HPV) vaccine, quadrivalent meningococcal conjugate vaccine (MenACWY), and tetanus and reduced diphtheria toxoids and acellular pertussis vaccine (Tdap). A booster dose of MenACWY is recommended at age 16 years (*1*), and catch-up vaccination is recommended for hepatitis B vaccine (HepB), measles, mumps, and rubella vaccine (MMR), and varicella vaccine (VAR) for adolescents whose childhood vaccinations are not up to date (UTD) (*1*). ACIP also recommends that clinicians may administer a serogroup B meningococcal vaccine (MenB) series to adolescents and young adults aged 16–23 years, with a preferred age of 16–18 years (*2*). To estimate U.S. adolescent vaccination coverage, CDC analyzed data from the 2017 National Immunization Survey–Teen (NIS-Teen) for 20,949 adolescents aged 13–17 years.* During 2016–2017, coverage increased for ≥1 dose of HPV vaccine (from 60.4% to 65.5%), ≥1 dose of MenACWY (82.2% to 85.1%), and ≥2 doses of MenACWY (39.1% to 44.3%). Coverage with Tdap remained stable at 88.7%. In 2017, 48.6% of adolescents were UTD with the HPV vaccine series (HPV UTD) compared with 43.4% in 2016.^†^ On-time vaccination (receipt of ≥2 or ≥3 doses of HPV vaccine by age 13 years) also increased. As in 2016, ≥1-dose HPV vaccination coverage was lower among adolescents living in nonmetropolitan statistical areas (MSAs) (59.3%) than among those living in MSA principal cities (70.1%).^§^ Although HPV vaccination initiation remains lower than coverage with MenACWY and Tdap, HPV vaccination coverage has increased an average of 5.1 percentage points annually since 2013, indicating that continued efforts to target unvaccinated teens and eliminate missed vaccination opportunities might lead to HPV vaccination coverage levels comparable to those of other routinely recommended adolescent vaccines.

NIS-Teen is an annual survey that estimates vaccination coverage among adolescents aged 13–17 years in the 50 states, the District of Columbia (DC), selected local areas, and territories.^¶^ NIS-Teen is conducted among parents and guardians of eligible adolescents identified using a random-digit–dialed sample of landline and cellular telephone numbers.** Parents and guardians are interviewed by telephone about the sociodemographic characteristics of the adolescent and household. Contact information and consent to contact the teen’s vaccination providers are requested. When more than one age-eligible adolescent lives in the household, one is randomly selected for participation. Vaccination providers identified during the interview are mailed a questionnaire requesting the vaccination history from the teen’s medical record.^††^ Vaccination coverage estimates are based on provider-reported vaccination histories. This report summarizes national vaccination coverage for 20,949 adolescents (9,845 females [47%] and 11,104 males [53%]) aged 13–17 years with adequate provider data.^§§^

Data were weighted and analyzed to account for the complex sampling design of NIS-Teen. NIS-Teen methodology, including methods for weighting and synthesizing provider-reported vaccination histories, has been described previously (*3*). T-tests were used to assess vaccination coverage differences between 2017 and 2016 and between demographic subgroups (i.e., age, health insurance status, MSA status, race/ethnicity, and poverty level). Weighted linear regression by survey year was used to estimate annual percentage point changes in coverage. Trends in HPV vaccination initiation and HPV UTD status by year of birth were assessed using combined data from 2016 and 2017 NIS-Teen; p-values <0.05 were considered statistically significant.

## National Vaccination Coverage

In 2017, coverage with ≥1 dose of HPV vaccine was 65.5% among teens, an increase of 5.1 percentage points compared with 2016; 48.6% were HPV UTD with the recommended vaccination series, an increase of 5.2 percentage points from 2016 (Table 1) (Figure). Among adolescents surveyed during 2016–2017, HPV vaccination initiation by age 13 years increased an average of 5.9 percentage points for each birth year, from 19.6% (1998 birth cohort) to 56.3% (2004 birth cohort) (Supplementary Figure 1, https://stacks.cdc.gov/view/cdc/58071). HPV UTD status by age 13 years increased an average of 3.6 percentage points for each birth year, from 7.7% (1998 birth cohort) to 29.8% (2004 birth cohort). Coverage with ≥1 and ≥2 MenACWY doses, ≥2 MMR doses, and ≥2 VAR doses also increased (Table 1). Coverage with ≥1 dose of MenB among persons aged 17 years was 14.5% (95% confidence interval [CI] = 12.3%–17.1%).

**TABLE 1:** Estimated vaccination coverage with selected vaccines and doses among adolescents aged 13–17* years, by age at interview –– National Immunization Survey–Teen (NIS–Teen), United States, 2017

Vaccine	Age (yrs), % (95% CI)^†^					Total, % (95% CI)^†^	
	13 (n = 4,283)	14 (n = 4,429)	15 (n = 4,212)	16 (n = 4,218)	17 (n = 3,807)	2017 (n = 20,949)	2016 (n = 20,475)
**Tdap^§^ ≥1 dose**	86.4 (84.0–88.4)	89.9 (88.0–91.5)^¶^	89.4 (87.7–91.0)^¶^	89.7 (87.7–91.5)^¶^	88.1 (85.4–90.3)	**88.7 (87.8–89.6)**	**88.0 (87.1–88.9)**
MenACWY**							
≥1 dose	83.6 (81.2–85.8)	85.8 (83.8–87.6)	85.1 (83.1–86.9)	86.6 (84.5–88.4)	84.4 (81.7–86.8)	**85.1 (84.2–86.1)^††^**	**82.2 (81.2–83.2)**
≥2 doses^§§^	NA	NA	NA	NA	44.3 (41.4–47.2)	**44.3 (41.4–47.2)^††^**	**39.1 (36.1–42.1)**
**HPV^¶¶^ vaccine – all adolescents**							
≥1 dose	60.7 (57.9–63.5)***	65.1 (62.5–67.6)^¶^	66.5 (63.8–69.1)^¶^	67.3 (64.7–69.8)^¶^	68.1 (65.4–70.7)^¶^	**65.5 (64.3–66.7)^††^**	**60.4 (59.2–61.6)**
UTD^†††^	39.0 (36.2–41.8)***	48.3 (45.5–51.2)^¶^	50.7 (47.8–53.6)^¶^	52.7 (49.8–55.5)^¶^	52.5 (49.5–55.4)^¶^	**48.6 (47.3–49.9)^††^**	**43.4 (42.1–44.7)**
**HPV^¶¶^ vaccine – females**							
≥1 dose	64.5 (60.5–68.3)***	67.8 (63.8–71.6)	67.2 (63.4–70.9)	71.5 (67.8–75.0)^¶^	72.0 (68.1–75.6)^¶^	**68.6 (66.9–70.2)^††^**	**65.1 (63.3–66.8)**
UTD	43.7 (39.6–47.8)***	52.7 (48.3–57.1)^¶^	53.3 (49.1–57.5)^¶^	57.5 (53.3–61.5)^¶^	58.7 (54.2–63.1)^¶^	**53.1 (51.2–55.0)^††^**	**49.5 (47.6–51.4)**
**HPV^¶¶^ vaccine – males**							
≥1 dose	57.1 (53.1–61.0)	62.4 (59.1–65.6)^¶^	65.7 (61.9–69.3)^¶^	63.4 (59.7–67.0)^¶^	64.3 (60.6–67.9)^¶^	**62.6 (60.9–64.2)^††^**	**56.0 (54.3–57.7)**
UTD	34.4 (30.8–38.2)	44.1 (40.6–47.6)^¶^	48.1 (44.1–52.2)^¶^	48.2 (44.3–52.1)^¶^	46.4 (42.5–50.4)^¶^	**44.3 (42.6–46.0)^††^**	**37.5 (35.8–39.2)**
**MMR ≥2 doses**	93.7 (92.4–94.8)	91.6 (89.6–93.3)	92.1 (90.3–93.5)	91.6 (89.5–93.2)	91.3 (89.4–92.9)^¶^	**92.1 (91.3–92.8)^††^**	**90.9 (90.1–91.6)**
**Hepatitis B vaccine ≥3 doses**	93.0 (91.4–94.3)	92.4 (90.6–93.8)	91.6 (89.8–93.1)	90.9 (88.9–92.6)	91.7 (89.8–93.3)	**91.9 (91.1–92.6)**	**91.4 (90.7–92.1)**
**Varicella vaccine**							
History of varicella disease^§§§^	9.8 (8.2–11.7)	11.4 (10.0–13.1)	13.7 (11.6–16.1)^¶^	14.9 (12.7–17.4)^¶^	16.5 (14.6–18.6)^¶^	**13.2 (12.3–14.2)^††^**	**15.2 (14.3–16.1)**
No history of varicella disease							
≥1 vaccine dose	96.7 (95.6–97.5)	95.7 (93.9–97.1)	95.5 (94.2–96.6)	94.4 (92.2–96.0)^¶^	94.9 (92.8–96.5)	**95.5 (94.8–96.1)**	**95.0 (94.2–95.6)**
≥2 vaccine doses	92.0 (90.2–93.6)	90.2 (87.9–92.1)	88.8 (86.6–90.7)^¶^	86.1 (83.7–88.2)^¶^	85.4 (82.7–87.7)^¶^	**88.6 (87.6–89.5)^††^**	**85.6 (84.5–86.6)**
History of varicella disease or receipt of ≥2 varicella vaccine doses	92.8 (91.1–94.2)	91.3 (89.2–93.0)	90.3 (88.4–92.0)^¶^	88.2 (86.1–90.0)^¶^	87.8 (85.5–89.7)^¶^	**90.1 (89.3–90.9)^††^**	**87.8 (86.9–88.6)**

**Abbreviations:** CI = confidence interval; HPV = human papillomavirus; MenACWY = quadrivalent meningococcal conjugate vaccine; MMR = measles, mumps, and rubella vaccine; NA = not applicable, Tdap = tetanus toxoid, reduced diphtheria toxoid, and acellular pertussis vaccine; UTD = up to date.

*Adolescents (N = 20,949) in the 2017 NIS–Teen were born January 1999 through February 2005.

^†^ Estimates with 95% CIs >20 might be unreliable.

^§^ Includes percentages receiving Tdap vaccine at age ≥10 years.

^¶^ Statistically significant difference (p<0.05) in estimated vaccination coverage by age; reference group was adolescents aged 13 years.

** Includes percentages receiving MenACWY or meningococcal vaccine of unknown type.

^††^ Statistically significant difference (p<0.05) compared with 2016 NIS-Teen estimates.

^§§^ ≥2 doses of MenACWY or meningococcal vaccine of unknown type. Calculated only among adolescents who were aged 17 years at interview. Does not include adolescents who received one dose of MenACWY vaccine at age ≥16 years.

^¶¶^ HPV vaccine, nine–valent (9vHPV), quadrivalent (4vHPV), or bivalent (2vHPV). For ≥1-dose measure, percentages are reported among females and males combined (N = 20,949) and for females only (N = 9,845) and males only (N = 11,104).

*** Statistically significant difference (p<0.05) in estimated vaccination coverage at age 13 years compared with 2016 NIS-Teen estimates.

^†††^ HPV UTD includes those with ≥3 doses, and those with 2 doses when the first HPV vaccine dose was initiated at age <15 years and at least 5 months minus 4 days elapsed between the first and second dose. This update to the HPV recommendation occurred in December of 2016.

^§§§^ By parent/guardian report or provider records.

**FIGURE:**
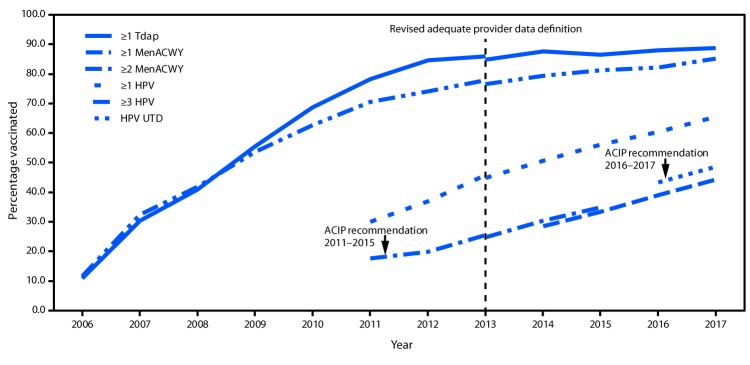
Estimated coverage with selected vaccines and doses* among adolescents aged 13–17 years, by survey year and ACIP recommendations^†^ — National Immunization Survey-Teen, United States, 2006–2017^§^ **Abbreviations:** ACIP = Advisory Committee on Immunization Practices; HPV = human papillomavirus; MenACWY = quadrivalent meningococcal conjugate vaccine; Tdap = tetanus toxoid, reduced diphtheria toxoid, and acellular pertussis vaccine; UTD = up to date. * ≥1 dose Tdap at or after age 10 years; ≥1 dose MenACWY or meningococcal-unknown type vaccine; ≥2 doses MenACWY or meningococcal-unknown type vaccine, calculated only among adolescents aged 17 years at time of interview. Does not include adolescents who received their first and only dose of MenACWY at or after 16 years of age; HPV vaccine, nine-valent (9vHPV), quadrivalent (4vHPV), or bivalent (2vHPV). The routine ACIP recommendation for HPV vaccination was made for females in 2006 and for males in 2011. Because HPV vaccination was not recommended for males until 2011, coverage for all adolescents was not measured before that year; HPV UTD includes those with ≥3 doses and those with 2 doses when the first HPV vaccine dose was initiated before age 15 years and at least 5 months minus 4 days elapsed between the first and second dose. ^†^ ACIP revised the recommended HPV vaccination schedule in late 2016. The recommendation changed from a 3-dose to 2-dose series with appropriate spacing between receipt of the first and second dose for immunocompetent adolescents initiating the series before the 15th birthday. Three doses are still recommended for adolescents initiating the series between the ages of 15 and 26 years. Because of the change in recommendation, the graph includes estimates for ≥3 doses HPV from 2011 to 2015 and the HPV UTD estimate for 2016 and 2017. Because HPV vaccination was recommended for boys in 2011, coverage for all adolescents was not measured before that year. ^§^ NIS-Teen implemented a revised adequate provider data definition (APD) in 2014, and retrospectively applied the revised APD definition to 2013 data. Estimates using different APD definitions may not be directly comparable.

## Vaccination Coverage by Selected Characteristics

Coverage with ≥1 dose of HPV vaccine and HPV UTD status were higher among adolescents living below the federal poverty level (73.3% and 53.7%, respectively) than among those living at or above the poverty level (62.8% and 46.7%, respectively)^¶¶^ (Table 2). Coverage with ≥1 dose of HPV vaccine was 10.8 percentage points lower among adolescents living in non-MSAs and 7.0 percentage points lower among those living in MSA nonprincipal cities compared with those living in MSA principal cities (Table 2). These disparities remained after controlling for poverty level.*** HPV UTD status was 10.0 percentage points lower among adolescents living in non-MSAs and 5.5 percentage points lower among those living in MSA nonprincipal cities compared with those living in MSA principal cities (Table 2). After adjusting for poverty level, differences in HPV UTD status did not persist among adolescents living in MSA nonprincipal cities, but did among adolescents living in non-MSAs compared with those living in MSA principal cities.^†††^ ≥1- and ≥2-dose MenACWY coverage rates among adolescents living in non-MSAs were 7.4 and 12.0 percentage points lower, respectively, than those among adolescents living in MSA principal cities (Table 2). This disparity remained after controlling for poverty level.^§§§^ Differences in HPV vaccination coverage by race/ethnicity in 2017 were similar to patterns observed in previous years (Supplementary Table 1, https://stacks.cdc.gov/view/cdc/58073) (*4*). Coverage with ≥1 dose of HPV vaccine and HPV UTD status were 8.8 and 6.6 percentage points higher, respectively, among adolescents enrolled in Medicaid than among those with private insurance only (Supplementary Table 2, https://stacks.cdc.gov/view/cdc/58074). HPV UTD status, ≥1-dose MenACWY, and ≥2-dose MenACWY coverage rates were 12.7, 5.0, and 22.6 percentage points lower, respectively, among uninsured adolescents than among those with private insurance (Supplementary Table 2).

**TABLE 2:** Estimated vaccination coverage with selected vaccines and doses among adolescents aged 13–17 years,* by poverty level^†^ and metropolitan statistical area^§^ — National Immunization Survey–Teen (NIS-Teen), United States, 2017

Vaccine	Poverty status % (95% CI)^¶^			Metropolitan statistical area (MSA) % (95% CI)^¶^				
	Below poverty level (n = 3,579)	At or above poverty level (n = 16,591)	Difference (n = 20,170)	Non-MSA (n = 4,123)	MSA nonprincipal city (n = 8,282)	MSA principal city (n = 8,544)	Difference between non-MSA and MSA principal city (n = 12,667)	Difference between MSA nonprincipal city and principal city (n = 16,826)
Tdap** ≥1 dose	88.2 (85.7 to 90.4)	88.8 (87.7 to 89.7)	-0.6 (-3.0 to 2.0)	88.0 (86.0 to 89.8)	88.9 (87.5 to 90.1)	88.8 (87.2 90.1)	-0.8 (-3.1 to 1.6)	0.1 (-1.8 to 2.1)
**MenACWY^††^**								
≥1 dose	85.7 (83.2 to 87.8)	84.8 (83.7 to 85.8)	0.9 (-1.7 to 3.4)	78.6 (76.3 to 80.7)^§§^	86.1 (84.6 to 87.4)	86.0 (84.4 to 87.4)	-7.4 (-10.0 to 4.7)^§§^	0.1 (-81.2 to 83.2)
≥2 doses^¶¶^	46.2 (38.6 to 54.0)	42.8 (39.7 to 45.9)	3.4 (-4.9 to 11.7)	35.0 (29.6 to 40.8)^§§^	44.3 (40.2 to 48.5)	47.0 (42.2 to 51.9)	-12.0 (-19.5 to 4.6)^§§^	-2.7 (-9.1 to 3.7)
HPV***								
≥1 dose	73.3 (70.7 to 75.8)^§§^	62.8 (61.4 to 64.1)	10.5 (7.6 to 13.5)^§§^	59.3 (56.6 to 61.9)^§§^	63.1 (61.3 to 64.8)^§§^	70.1 (68.2 to 71.9)	-10.8 (-14.0 to 7.6)^§§^	-7.0 (-9.6 to 4.4)^§§^
UTD^†††^	53.7 (50.7 to 56.6)^§§^	46.7 (45.3 to 48.2)	7.0 (3.6 to 10.3)^§§^	42.4 (39.8 to 45.1)^§§^	46.9 (45.0 to 48.8)^§§^	52.4 (50.3 to 54.4)	-10.0 (-13.3 to 6.6)^§§^	-5.5 (-8.3 to 2.6)^§§^
**≥2 MMR doses**	90.6 (88.4 to 92.5)	92.4 (91.5 to 93.1)	-1.8 (-3.9 to 0.5)	92.0 (90.6 to 93.3)	92.1 (90.9 to 93.1)	92.1 (90.7 to 93.3)	-0.1 (-1.9 to 1.8)	0.0 (-1.7 to 1.7)
**≥3 Hepatitis B doses**	89.9 (87.6 to 91.8)^§§^	92.5 (91.7 to 93.3)	-2.6 (-4.8 to 0.3) ^§§^	91.3 (89.6 to 92.7)	92.0 (90.9 to 93.0)	92.0 (90.6 to 93.1)	-0.7 (-2.7 to 1.3)	0.0 (-1.6 to 1.7)
**Varicella**								
History of varicella disease^§§§^	13.8 (12.1 to 15.6)	12.6 (11.6 to 13.6)	1.2 (-0.8 to 3.2)	16.1 (14.2 to 18.2)	12.2 (11.0 to 13.5)	13.6 (12.1 to 15.2)	2.5 (0.0 to 5.1)	-1.4 (-3.4 to 0.6)
**No history of varicella disease**								
≥1 varicella vaccine dose	94.4 (91.9 to 96.1)	95.7 (95.0 to 96.4)	-1.3 (-3.5 to 0.8)	95.4 (94.1 to 96.5)	95.6 (94.6 to 96.5)	95.4 (94.0 to 96.4)	0.0 (-1.6 to 1.7)	0.2 (-1.3 to 1.8)
≥2 varicella vaccine doses	88.2 (85.5 to 90.4)	88.6 (87.6 to 89.6)	-0.4 (-3.1 to 2.2)	87.3 (85.4 to 89.1)	88.8 (87.4 to 90.1)	88.7 (87.0 to 90.2)	-1.4 (−3.8 to 1.1)	0.1 (-1.9 to 2.3)
**History of varicella or receipt of ≥2 doses varicella vaccine**	89.8 (87.5 to 91.7)	90.1 (89.1 to 90.9)	-0.3 (-2.6 to 2.0)	89.4 (87.7 to 90.8)	90.2 (88.9 to 91.3)	90.2 (88.7 to 91.5)	-0.8 (-2.9 to 1.3)	0.0 (-1.8 to 1.9)

**Abbreviations:** CI = confidence interval; HPV = human papillomavirus; MenACWY = quadrivalent meningococcal conjugate vaccine; MMR = measles, mumps, and rubella vaccine; Tdap = tetanus toxoid, reduced diphtheria toxoid, and acellular pertussis vaccine; UTD = up-to-date.

* Adolescents (N = 20,949) in the 2017 NIS-Teen were born January 1999 through February 2005.

^†^ Adolescents were classified as below poverty level if their total family income was less than the federal poverty level specified for the applicable family size and number of children aged <18 years. All others were classified as at or above the poverty level. Additional information available at https://www.census.gov/data/tables/time-series/demo/income-poverty/historical-poverty-thresholds.html. Poverty status was unknown for 779 adolescents.

^§^ MSA status was determined based on household-reported county and city of residence, and was grouped into three categories: MSA principal city, MSA nonprincipal city, and non-MSA. MSA and principal city were as defined by the U.S. Census Bureau (https://www.census.gov/geo/reference/gtc/gtc_cbsa.html). Non-MSA areas include urban populations not located within an MSA as well as completely rural areas.

^¶^ Estimates with 95% CIs >20 might be unreliable.

** Includes percentages receiving Tdap vaccine at age ≥10 years.

^††^ Includes percentages receiving MenACWY and meningococcal vaccine of unknown type.

^§§^ Statistically significant difference (p<0.05) in estimated vaccination coverage by poverty level or metropolitan statistical area; the referent groups were adolescents living at or above poverty level and MSA principal city respectively.

^¶¶^ ≥2 doses of MenACWY or meningococcal vaccine of unknown type vaccine. Calculated only among adolescents aged 17 years at interview. Does not include adolescents who received one dose of MenACWY vaccine at age ≥16 years.

*** HPV vaccine, nine-valent (9vHPV), quadrivalent (4vHPV), or bivalent (2vHPV) in females and males combined.

^†††^ HPV UTD includes those with ≥3 doses and those with 2 doses when the first HPV vaccine dose was initiated at age <15 years and at least 5 months minus 4 days elapsed between the first and second dose. This update to the HPV recommendation occurred in December of 2016.

^§§§^ By parent/guardian report or provider records.

## State, Local, and Territorial Vaccination Coverage

Vaccination coverage varied by jurisdiction (Table 3). Coverage with ≥1 dose of Tdap ranged from 78.9% in Alaska to 96.2% in Massachusetts; with ≥1 dose of MenACWY, from 60.7% in Wyoming to 95.3% in Georgia; and with ≥1 dose of HPV vaccine, from 46.9% in Wyoming to 91.9% in DC (Table 3) (Supplementary Figure 2, https://stacks.cdc.gov/view/cdc/58072). HPV UTD status ranged from 28.8% in Mississippi to 78.0% in DC. The largest increases in HPV UTD status from 2016 to 2017 occurred in Virginia (19.8 percentage points), DC (16.0), South Carolina (13.6), Nebraska (12.4), Dallas, Texas (11.8), Louisiana (11.1), North Carolina (10.7), Massachusetts (8.9), Vermont (8.8), and Texas (6.8) (Table 3). During 2013–2017, ≥1-dose HPV vaccination coverage increased an average of 5.1 percentage points per year nationally; the 5-year average annual increase ranged from 2.2 to 8.5 percentage points. The largest average annual increases were in Virginia (8.5 percentage points), DC (7.5), Montana (7.4), and in Arkansas, Iowa, Utah, and El Paso, Texas (7.3 percentage points each) (Supplementary Table 3, https://stacks.cdc.gov/view/cdc/58075).

**TABLE 3:** Estimated vaccination coverage with selected vaccines and doses* among adolescents aged 13–17 years,^†^ by HHS region, state, selected local area, or territory — National Immunization Survey–Teen (NIS-Teen), United States, 2017

Region, state, local area	All adolescents (N = 20,949) % (95% CI)^§^			
	≥1 Tdap^¶^	≥1 MenACWY**	≥1 HPV^††^	HPV UTD^§§^
**United States overall**	**88.7 (87.8–89.6)**	**85.1 (84.2–86.1)^¶¶^**	**65.5 (64.3–66.7)^¶¶^**	**48.6 (47.3–49.9)^¶¶^**
**Region I**	**94.6 (93.1–95.7)**	**92.5 (90.8–93.9)**	**78.2 (75.4–80.8)^¶¶^**	**63.3 (60.1–66.4)^¶¶^**
Connecticut	94.9 (91.9–96.8)	94.9 (91.4–97.0)	71.3 (64.9–76.9)^¶¶^	58.0 (51.4–64.3)
Maine	85.1 (79.8–89.3)	83.9 (78.8–88.0)	75.8 (70.2–80.6)	59.2 (53.2–65.0)
Massachusetts	96.2 (93.4–97.8)	94.0 (90.7–96.2)	81.9 (76.9–85.9)^¶¶^	65.5 (59.7–70.8)^¶¶^
New Hampshire	95.1 (91.6–97.2)	87.9 (82.9–91.6)	74.2 (68.5–79.2)	59.9 (53.7–65.8)
Rhode Island	94.6 (91.0–96.8)	94.1 (90.2–96.5)	88.6 (83.3–92.4)	77.7 (71.6–82.8)
Vermont	92.8 (89.2–95.2)	84.2 (78.9–88.3)	78.7 (73.2–83.3)^¶¶^	64.5 (58.4–70.2)^¶¶^
**Region II**	**91.9 (89.7–93.7)**	**90.6 (88.1–92.6)**	**67.6 (64.1–71.0)**	**52.3 (48.5–56.0)**
New Jersey	90.0 (85.3–93.3)	93.3 (89.4–95.9)	65.8 (59.8–71.3)	49.6 (43.4–55.8)
New York	92.9 (90.3–94.8)	89.3 (85.9–91.9)	68.5 (64.0–72.7)	53.6 (48.9–58.2)
New York - New York City	92.9 (89.0–95.5)	88.8 (83.6–92.6)	73.3 (66.9–78.9)	61.0 (54.1–67.5)
New York - rest of state	92.8 (89.1–95.4)	89.5 (84.9–92.9)	65.5 (59.3–71.2)	48.8 (42.6–55.0)
**Region III**	**89.5 (87.0–91.6)**	**88.8 (86.3–91.0)^¶¶^**	**70.3 (67.0–73.3)^¶¶^**	**54.5 (51.0–57.9)^¶¶^**
Delaware	89.6 (84.5–93.2)	90.5 (85.7–93.7)	75.3 (69.3–80.5)	58.1 (51.6–64.4)
District of Columbia	86.1 (80.2–90.4)	91.3 (85.7–94.9)	91.9 (87.6–94.8)^¶¶^	78.0 (71.1–83.6)^¶¶^
Maryland	88.3 (82.2–92.5)	91.8 (86.5–95.1)^¶¶^	69.2 (62.1–75.6)	52.9 (45.4–60.2)
Pennsylvania	90.6 (86.7–93.5)	93.4 (90.3–95.6)	67.3 (62.2–72.1)	52.5 (47.3–57.7)
Pennsylvania - Philadelphia	91.6 (87.4–94.5)	91.1 (86.8–94.1)	84.9 (80.0–88.7)	69.5 (63.5–75.0)
Pennsylvania - rest of state	90.5 (85.9–93.7)	93.7 (90.1–96.0)	65.0 (59.2–70.4)	50.3 (44.5–56.0)
Virginia	89.3 (83.2–93.4)	80.0 (72.6–85.7)	75.6 (68.4–81.6)^¶¶^	59.0 (51.1–66.6)^¶¶^
West Virginia	87.5 (82.8–91.0)	87.9 (83.1–91.5)	60.9 (54.6–66.9)	43.9 (37.7–50.2)
**Region IV**	**90.9 (89.3–92.2)**	**82.2 (80.0–84.1)^¶¶^**	**60.0 (57.3–62.6)^¶¶^**	**43.0 (40.3–45.7)^¶¶^**
Alabama	88.7 (84.3–92.0)	78.3 (73.0–82.9)	58.0 (52.0–63.9)	40.3 (34.4–46.5)
Florida	91.1 (87.1–94.0)	80.2 (74.3–85.0)	59.8 (53.1–66.1)	42.3 (35.9–49.0)
Georgia	93.3 (89.3–95.9)	95.3 (91.9–97.3)	64.3 (57.5–70.6)	45.7 (39.1–52.5)
Kentucky	86.4 (81.7–90.0)	83.3 (78.3–87.4)	49.6 (43.5–55.6)	37.7 (32.1–43.7)
Mississippi	92.4 (88.6–95.0)^¶¶^	63.0 (56.9–68.7)	49.6 (43.4–55.9)	28.8 (23.5–34.8)
North Carolina	91.9 (87.8–94.7)	84.8 (79.4–89.0)^¶¶^	66.8 (60.4–72.6)^¶¶^	51.9 (45.3–58.4)^¶¶^
South Carolina	89.4 (84.5–92.8)^¶¶^	78.6 (72.4–83.7)^¶¶^	59.6 (52.7–66.0)^¶¶^	42.7 (36.1–49.5)^¶¶^
Tennessee	89.4 (84.8–92.8)	75.0 (68.5–80.6)	56.1 (49.3–62.6)	39.2 (32.8–46.1)
**Region V**	**91.8 (90.4–93.0)**	**89.4 (87.8–90.7)^¶¶^**	**65.5 (63.2–67.8)^¶¶^**	**49.0 (46.5–51.4)^¶¶^**
Illinois	92.4 (89.4–94.6)	89.2 (85.9–91.8)^¶¶^	66.1 (61.5–70.4)	50.4 (45.8–55.0)
Illinois - Chicago	90.5 (84.9–94.2)	90.9 (83.4–95.2)	81.9 (73.9–87.8)	66.6 (57.7–74.4)
Illinois - rest of state	92.8 (89.2–95.3)	88.9 (85.0–91.8)^¶¶^	62.7 (57.4–67.7)	46.9 (41.7–52.2)
Indiana	95.1 (92.3–96.9)^¶¶^	93.1 (89.0–95.8)	59.3 (52.8–65.5)^¶¶^	40.8 (34.4–47.5)
Michigan	93.4 (89.2–96.0)	93.5 (89.4–96.1)	67.3 (61.1–73.0)	54.3 (47.9–60.6)
Minnesota	87.5 (82.2–91.4)	87.5 (82.4–91.3)	68.1 (61.9–73.7)^¶¶^	46.9 (40.7–53.3)
Ohio	90.6 (86.9–93.3)	87.3 (83.4–90.4)^¶¶^	64.1 (58.4–69.3)	47.0 (41.2–52.8)
Wisconsin	90.3 (85.8–93.5)	83.8 (78.4–88.2)	69.2 (63.0–74.8)	52.3 (45.8–58.7)
**Region VI**	**85.0 (83.0–86.8)**	**84.4 (82.4–86.2)**	**59.7 (57.1–62.2)^¶¶^**	**41.3 (38.9–43.8)^¶¶^**
Arkansas	92.4 (88.6–94.9)	91.7 (87.4–94.7)	61.1 (54.8–67.0)	35.2 (29.4–41.5)
Louisiana	90.1 (85.5–93.4)	89.0 (84.3–92.5)	69.1 (63.3–74.4)^¶¶^	52.9 (46.5–59.1)^¶¶^
New Mexico	85.5 (80.3–89.5)	78.0 (72.4–82.8)	66.9 (60.9–72.4)	48.3 (42.2–54.5)
Oklahoma	86.7 (81.7–90.5)	71.1 (64.9–76.6)	58.5 (52.1–64.6)	41.4 (35.3–47.8)
Texas	83.2 (80.4–85.7)	85.1 (82.4–87.5)	57.8 (54.3–61.2 ^¶¶^	39.7 (36.5–43.0)^¶¶^
Texas - Bexar County	83.7 (77.8–88.3)	86.0 (80.3–90.3)	62.9 (56.6–68.8)^¶¶^	46.4 (40.2–52.7)
Texas - Houston	87.9 (80.2–92.9)	91.4 (85.1–95.2)	73.0 (63.9–80.4)	55.2 (45.9–64.2)
Texas - Dallas County	77.0 (69.8–83.0)	85.1 (78.8–89.7)	54.5 (46.9–62.0)	35.7 (28.8–43.1)^¶¶^
Texas - El Paso County	89.6 (84.8–93.0)	89.5 (84.4–93.0)	82.8 (77.2–87.2)	60.0 (52.9–66.6)
Texas - Travis County	85.9 (80.9–89.8)	89.1 (84.4–92.4)	69.7 (63.3–75.4)	52.0 (45.4–58.5)
Texas - rest of state	83.1 (79.3–86.3)	84.1 (80.4–87.2)	54.5 (49.9–59.1)^¶¶^	36.6 (32.4–41.0)
**Region VII**	**86.8 (84.0–89.2)**	**77.3 (74.2–80.2)^¶¶^**	**61.5 (58.0–64.8)^¶¶^**	**44.2 (40.9–47.6)**
Iowa	93.4 (89.8–95.8)	83.6 (78.4–87.7)^¶¶^	71.4 (65.6–76.5)^¶¶^	53.7 (47.6–59.8)
Kansas	89.7 (84.9–93.1)	72.1 (65.8–77.6)	52.4 (46.0–58.8)	34.4 (28.6–40.7)
Missouri	80.1 (74.1–85.0)	74.3 (68.3–79.5)	57.8 (51.3–64.0)	39.6 (33.6–45.9)
Nebraska	92.3 (87.5–95.4)	84.8 (79.4–89.0)	71.0 (64.8–76.5)	58.3 (51.9–64.5)^¶¶^
**Region VIII**	**89.1 (86.6–91.1)**	**81.4 (78.7–83.8)^¶¶^**	**65.7 (62.4–68.8)^¶¶^**	**46.8 (43.4–50.3)^¶¶^**
Colorado	88.6 (83.6–92.2)	82.4 (77.2–86.6)	72.1 (66.2–77.3)	53.8 (47.4–60.0)
Montana	90.4 (85.8–93.7)	71.2 (64.9–76.8)	65.5 (58.9–71.5)^¶¶^	49.1 (42.5–55.7)
North Dakota	90.6 (86.8–93.5)	91.9 (88.3–94.4)	72.5 (67.0–77.4)	57.8 (51.9–63.5)
South Dakota	79.5 (73.6–84.4)	74.5 (68.4–79.9)^¶¶^	63.2 (56.7–69.2)	44.8 (38.5–51.2)
Utah	91.6 (87.7–94.3)^¶¶^	85.1 (80.3–88.9)^¶¶^	58.8 (52.6–64.8)	37.4 (31.5–43.7)
Wyoming	86.4 (81.2–90.3)	60.7 (54.5–66.6)	46.9 (40.8–53.1)	30.9 (25.5–36.8)
**Region IX**	**83.3 (78.5–87.2)**	**82.2 (77.4–86.2)**	**70.4 (65.4–75.0)**	**53.1 (47.5–58.7)**
Arizona	82.4 (76.7–87.0)	83.8 (78.3–88.1)	65.0 (58.4–71.2)	53.0 (46.3–59.6)
California	83.5 (77.2–88.3)	82.2 (75.9–87.1)	71.9 (65.4–77.5)	53.4 (46.3–60.4)
Hawaii	84.8 (79.3–89.1)	85.9 (80.6–90.0)^¶¶^	69.4 (63.0–75.1)	54.7 (48.2–61.0)
Nevada	82.5 (76.6–87.1)	77.3 (71.0–82.5)	64.9 (58.3–70.9)	49.0 (42.6–55.5)
**Region X**	**87.2 (84.5–89.5)**	**81.4 (78.2–84.2)^¶¶^**	**69.9 (66.3–73.3)^¶¶^**	**52.8 (48.9–56.6)^¶¶^**
Alaska	78.9 (73.2–83.6)	68.4 (62.5–73.8)	64.5 (58.4–70.1)	42.6 (36.7–48.8)
Idaho	87.3 (82.1–91.1)	90.5 (85.6–93.9)	62.4 (55.7–68.6)	44.1 (37.6–50.7)
Oregon	86.3 (81.6–90.0)	77.0 (71.5–81.8)	71.2 (65.4–76.4)^¶¶^	54.8 (48.6–60.8)
Washington	88.6 (83.8–92.1)	82.6 (77.2–87.0)	71.9 (65.8–77.3)	55.2 (48.8–61.5)
Range***	**(78.9–96.2)**	**(60.7–95.3)**	**(46.9–91.9)**	**(28.8–78.0)**
**Territory**				
Guam	77.3 (71.6–82.1)	68.3 (62.2–73.9) **^†††^**	67.5 (61.4–73.0)	42.7 (36.9–48.8)

**Abbreviations:** CI = confidence interval; HHS = U.S. Department of Health and Human Services; HPV = human papillomavirus; MenACWY = quadrivalent meningococcal conjugate vaccine; MMR = measles, mumps, rubella vaccine; Tdap = tetanus toxoid, reduced diphtheria toxoid, and acellular pertussis vaccine; UTD = up-to-date.

* Estimates for additional measures, including MMR, hepatitis B, and varicella vaccines are available at https://www.cdc.gov/vaccines/vaxview/teenvaxview.

^†^ Adolescents (N = 20,949) in the 2017 NIS-Teen were born January 1999 through February 2005.

^§^ Estimates with 95% CIs >20 might be unreliable.

^¶^ ≥1 dose Tdap vaccine at age ≥10 years.

** ≥1 dose of MenACWY or meningococcal-unknown type vaccine.

^††^ HPV vaccine, nine-valent (9vHPV), quadrivalent (4vHPV), or bivalent (2vHPV) in females and males combined.

^§§^ HPV UTD includes those with ≥3 doses and those with 2 doses when the first HPV vaccine dose was initiated before age 15 years and there was at least 5 months minus 4 days between the first and second dose. This update to the HPV recommendation occurred in December of 2016.

^¶¶^ Statistically significant (p<0.05) percentage point increase compared to 2016.

*** The calculation for the range was limited to the 50 states and the District of Columbia.

^†††^ Statistically significant (p<0.05) percentage point decrease from 2016.

## Discussion

In 2017, adolescent vaccination coverage with ≥1 dose of HPV vaccine, ≥1 and ≥2 doses of MenACWY, ≥2 doses of MMR, and ≥2 doses of VAR increased, while coverage with ≥1 dose of Tdap and ≥3 doses of HepB remained high. This report includes the first U.S. estimates of ≥1-dose MenB coverage. Unlike MenACWY, MenB is not routinely recommended for all adolescents, and thus, the low vaccination coverage in adolescents aged 17 years (14.5%) is not unexpected.

In December 2016, a 2-dose HPV vaccine schedule was recommended for persons starting the series at age <15 years, based on data showing noninferior immunogenicity compared with 3 doses (*5*). This schedule might encourage on-time initiation of the series and facilitate completion; however, it is too early to assess its impact on vaccination coverage. The 5.1 percentage point annual increase in series initiation among all adolescents since 2013 is encouraging. Moreover, on-time vaccination (series completion by age 13 years) has increased approximately four percentage points in each successive birth cohort. Despite these improvements, HPV vaccination initiation remains lower than coverage with Tdap and MenACWY, suggesting ongoing challenges to providing all three vaccines during the same visit. Efforts are under way to promote and improve on-time vaccination, including implementing a new combined Healthcare Effectiveness Data and Information Set measure for adolescent vaccines that assesses receipt of all three routinely recommended adolescent vaccines, including HPV vaccine series completion by age 13 years (*6*).

HPV vaccine and MenACWY coverage in non-MSA areas remains lower than that in MSA areas. Disparities in coverage by MSA status were not observed for Tdap. Unlike persons living in urban settings, rural residents are less likely to have knowledge of HPV or be aware of HPV vaccine and its importance in cancer prevention (*7*,*8*). The overall shortage of health care providers, especially pediatricians, in rural areas might partially explain the lower coverage among rural adolescents (*8*,*9*). Health care providers in rural areas serve a broader population base and might be less familiar with adolescent vaccination recommendations. A study including adolescents and parents in rural Alabama identified provider education, better communication with parents and adolescents about the importance of HPV vaccination for preventing cancer, and a strong provider recommendation as being most influential in initiation of HPV vaccination (*7*). Resources are available to facilitate discussion with adolescents and their parents about the importance of HPV vaccination (https://www.cdc.gov/hpv/). Further evaluation is needed to identify where teens are receiving Tdap in non-MSAs and better understand the barriers to providing HPV vaccine and MenACWY at these sites.

The findings in this report are subject to at least five limitations. First, the overall household response rate was 25.7% (landline = 51.5%; cell phone = 23.5%), and only 53.6% of landline-completed and 47.1% of cell phone–completed interviews included adequate provider data. Second, bias in estimates might remain after adjustment for household and provider nonresponse and phoneless households.^¶¶¶^ Weights have been adjusted for the increasing number of cell phone–only households over time. Nonresponse bias might change, which could affect comparisons of estimates between survey years. Third, estimates stratified by state/local area might be unreliable because of small sample sizes. Fourth, multiple statistical tests were conducted, and a small number might be significant because of chance alone. Finally, because NIS-Teen includes adolescents aged 13–17 years, data on receipt of MenACWY or MenB vaccine at age ≥18 years could not be collected; thus reported coverage with these vaccines might underestimate the proportion of adolescents receiving them (*1*).

HPV vaccination initiation and completion continue to increase. Postintroduction monitoring studies have found reductions in cervical HPV infection, genital warts, and cervical precancers in the United States (*10*). Protection against HPV-related cancers will continue to increase if adolescents and their parents are educated about the cancer prevention benefits of HPV vaccine and clinicians consistently recommend and simultaneously administer Tdap, MenACWY, and HPV vaccine at age 11–12 years.

**Summary**

**What is already known about this topic?**

Vaccines to prevent human papillomavirus (HPV)–associated cancers, diphtheria, pertussis, tetanus, and meningococcal diseases are routinely recommended for persons aged 11–12 years.

**What is added by this report?**

In 2017, coverage among adolescents aged 13–17 years increased for ≥1 dose of HPV vaccine and ≥1 and ≥2 doses of meningococcal vaccines and remained high for ≥1 dose of tetanus and diphtheria toxoids and acellular pertussis vaccine. HPV vaccination initiation has increased an average of 5.1 percentage points annually since 2013.

**What are the implications for public health care?**

The increase in HPV vaccination coverage indicates that further efforts to address barriers to HPV vaccination initiation and series completion likely will lead to greater protection against HPV-associated cancers.

## Footnotes

* Eligible participants were born during January 1999–February 2005. Tdap coverage represents receipt of ≥1 Tdap dose at age ≥10 years. MenACWY coverage represents receipt of the quadrivalent meningococcal conjugate vaccine or meningococcal vaccine of unknown type. MenB coverage represents receipt of at least 1 dose of either a 2-dose or 3-dose series, depending upon the vaccine brand. HPV vaccination coverage includes receipt of any HPV vaccine and does not distinguish between nine-valent (9vHPV), quadrivalent (4vHPV), or bivalent (2vHPV) vaccines. Some adolescents might have received more than the 2 or 3 recommended HPV vaccine doses. Estimates for hepatitis B and MMR vaccines represent coverage based on catch up for adolescents who were not up-to-date with these vaccinations. Except as noted, coverage estimates for ≥1 and ≥2 varicella vaccine doses were obtained among adolescents with no history of varicella disease. Influenza vaccination coverage data are not included in this report but are available online at https://www.cdc.gov/flu/fluvaxview/index.htm.

† Adolescents were considered to be HPV UTD if they had received ≥3 doses, or if all of the following applied: 1) they had received 2 doses; 2) the first dose was received before the 15th birthday; and 3) the interval between the first and second doses was ≥5 months minus 4 days, the absolute minimum interval between the first and second doses. https://www.cdc.gov/vaccines/programs/iis/cdsi.html.

§ MSA status was determined based on household reported city and county of residence, and status was grouped into three categories: MSA principal city, MSA nonprincipal city, and non-MSA. MSA and principal city were as defined by the U.S. Census Bureau (https://www.census.gov/geo/reference/gtc/gtc_cbsa.html). Non-MSA areas include urban populations not located within an MSA as well as completely rural areas.

¶ The following local areas that received federal Section 317 immunization funds were sampled separately: Chicago, Illinois; New York, New York; Philadelphia County, Pennsylvania; Bexar County, Texas; and Houston, Texas. Three local areas were oversampled (Dallas County, Texas, El Paso County, Texas, and Travis County, Texas). Three territories were sampled separately in 2017 (Guam, Puerto Rico, and the U.S. Virgin Islands). Because of the severity of 2017’s hurricane season, survey operations in Puerto Rico and the U.S. Virgin Islands were suspended resulting in insufficient data for estimation of vaccination coverage.

** All identified cellular-telephone households were eligible for interview. Sampling weights were adjusted for dual-frame (landline and cellular telephone), nonresponse, noncoverage, and overlapping samples of mixed telephone users. A description of NIS-Teen dual-frame survey methodology and its effect on reported vaccination estimates is available at https://www.cdc.gov/vaccines/imz-managers/coverage/nis/child/dual-frame-sampling.html. Starting in 2018, the landline telephone sample was dropped.

†† For the telephone samples for the states and local areas, the overall Council of American Survey Research Organizations (CASRO) response rate was 25.7% (51.5% for the landline sample and 23.5% for the cellular-telephone sample). For adolescents with completed interviews, 48.1% had adequate provider data (53.6% landline sample, 47.1% cell sample). Among completed interviews with adequate provider data, 17% (3,572) were from the landline sample, and 83% (17,377) were from the cellular telephone sample. For Guam, the overall CASRO response rate was 31.3%. The CASRO response rate is the product of three other rates: 1) the resolution rate (the proportion of telephone numbers that can be identified as either for business or residence); 2) the screening rate (the proportion of qualified households that complete the screening process); and 3) the cooperation rate (the proportion of contacted eligible households for which a completed interview is obtained).

§§ Adolescents from Guam (n = 382).

¶¶ Adolescents were classified as below the federal poverty level if their total family income was less than the federal poverty level specified for the applicable family size and number of children aged <18 years. All others were classified as at or above the poverty level. Poverty status was unknown for 779 adolescents. https://www.census.gov/data/tables/time-series/demo/income-poverty/historical-poverty-thresholds.html.

*** Among adolescents living below poverty level, ≥1-dose HPV vaccination coverage estimates stratified by MSA status were 63.7% (95% CI = 58.4%–68.7%) for adolescents living in non-MSAs, 70.4% (CI = 65.8%–74.7%) for adolescents living in MSA nonprincipal cities, and 78.0% (CI = 74.1%–81.5%) for adolescents living in MSA principal cities (reference group). Among adolescents living at or above poverty level, ≥1-dose HPV vaccination coverage estimates were 56.9% (CI = 53.8%–60.0%) for adolescents living in non-MSAs, 61.6% (CI = 59.6%–63.6%) for adolescents living in MSA nonprincipal cities, and 66.0% (CI = 63.7%–68.2%) for adolescents living in MSA principal cities (reference group).

††† Among adolescents living below poverty level, HPV UTD status estimates stratified by MSA status were 44.3% (CI = 39.1%–49.6%), 52.8% (CI = 47.8%–57.8%), and 57.0% (CI = 52.4%–61.5%) for adolescents living in non-MSAs, MSA nonprincipal cities, and MSA principal cities (reference group), respectively. Among adolescents living at or above poverty level, HPV UTD status estimates stratified by MSA status were 40.7% (CI = 37.6%–48.3%), 46.1% (CI = 44.0%–48.2%), and 49.3% (CI = 46.9%–51.7%) for adolescents living in non-MSAs, MSA nonprincipal cities, and MSA principal cities (reference group), respectively.

§§§ Among adolescents living below poverty level, ≥1-dose MenACWY coverage estimates stratified by MSA status were 83.2% (CI = 79.3%–86.5%), 87.7% (CI = 84.0%–90.7%), and 85.1% (CI = 80.9%–88.5%) for adolescents living in non-MSAs, MSA nonprincipal cities, and MSA principal cities (reference group), respectively. Among adolescents living at or above poverty level, ≥1-dose MenACWY coverage estimates were 76.0% (CI = 73.1%–78.7%), 85.7% (CI = 84.0%–87.3%), and 86.0% (CI = 84.3%–87.5%) for adolescents living in non-MSAs, MSA nonprincipal cities, and MSA principal cities (reference group), respectively.

¶¶¶ In a sensitivity analysis of 2013 NIS-Teen data, including adjustments for incomplete sample frame, nonresponse bias, and incomplete ascertainment of vaccination status, estimates of Tdap, ≥1 dose MenACWY, and ≥1 dose HPV vaccine coverage, were estimated to be lower than actual values by 1–3 percentage points https://www.cdc.gov/vaccines/imz-managers/nis/downloads/NIS-TEEN-PUF16-DUG.pdf.
