# *Erratum:* Vol. 67, No. 33

**DOI:** 10.15585/mmwr.mm6741a8

**Published:** 2018-10-19

In the report “National, Regional, State, and Selected Local Area Vaccination Coverage Among Adolescents Aged 13–17 Years — United States, 2017,” on page 914, in Table 2, the entry for the column “Difference” and row “≥3 Hepatitis B doses” should have read -2.6 (-4.8 to **-0.3**). The entry for the column “MSA principal city” and row “Tdap ≥1 dose” should have read “88.8 (87.2 **to** 90.1). Entries for the column “Difference between non-MSA and MSA principal city” and row “MenACWY, ≥1 dose” should have read -7.4 (-10.0 to **-4.7**), row “MenACWY, ≥2 dose” should have read -12.0 (-19.5 to **-4.6**), row “HPV, ≥1dose” should have read -10.8 (-14.0 to **-7.6**), and row “HPV UTD” should have read -10.0 (-13.3 to **-6.6**). Entries for the column “Difference between MSA nonprincipal city and principal city” and row “MenACWY, ≥1 dose” should have read 0.1 (**-2.0 to 2.2**), row “HPV, ≥1dose” should have read -7.0 (­‑9.6 to **-4.4**), and row “HPV UTD” should have read -5.5 (-8.3 to **-2.6**).

